# Varanid Lizard Venoms Disrupt the Clotting Ability of Human Fibrinogen through Destructive Cleavage

**DOI:** 10.3390/toxins11050255

**Published:** 2019-05-07

**Authors:** James S. Dobson, Christina N. Zdenek, Chris Hay, Aude Violette, Rudy Fourmy, Chip Cochran, Bryan G. Fry

**Affiliations:** 1 Venom Evolution Lab, School of Biological Sciences, University of Queensland, St Lucia, QLD 4072, Australia; j.dobson@uq.edu.au (J.S.D.); christinazdenek@gmail.com (C.N.Z.); tropidechis@hotmail.com (C.H.); 2 Alphabiotoxine Laboratory sprl, Barberie 15, 7911 Montroeul-au-bois, Belgium; rd.science@alphabiotoxine.be (A.V.); info@alphabiotoxine.be (R.F.); 3 Department of Earth and Biological Sciences, Loma Linda University, Loma Linda, CA 92350, USA; skipc8384@hotmail.com

**Keywords:** Anguimorpha, *Varanus*, *Heloderma*, lizard venom, fibrinogenolytic, anticoagulation

## Abstract

The functional activities of Anguimorpha lizard venoms have received less attention compared to serpent lineages. Bite victims of varanid lizards often report persistent bleeding exceeding that expected for the mechanical damage of the bite. Research to date has identified the blockage of platelet aggregation as one bleeding-inducing activity, and destructive cleavage of fibrinogen as another. However, the ability of the venoms to prevent clot formation has not been directly investigated. Using a thromboelastograph (TEG5000), clot strength was measured after incubating human fibrinogen with *Heloderma* and *Varanus* lizard venoms. Clot strengths were found to be highly variable, with the most potent effects produced by incubation with *Varanus* venoms from the *Odatria* and *Euprepriosaurus* clades. The most fibrinogenolytically active venoms belonged to arboreal species and therefore prey escape potential is likely a strong evolutionary selection pressure. The results are also consistent with reports of profusive bleeding from bites from other notably fibrinogenolytic species, such as *V. giganteus*. Our results provide evidence in favour of the predatory role of venom in varanid lizards, thus shedding light on the evolution of venom in reptiles and revealing potential new sources of bioactive molecules useful as lead compounds in drug design and development.

## 1. Introduction

The widely accepted Toxicofera clade consists of the lizard clades Anguimorpha and Iguania, in addition to the Serpents, and encompasses all known venomous reptiles [1,2,3]. This clade has been consistently recovered by studies utilising genetic and integrated morphological/molecular evidence [2,4,5,6,7,8,9]. Despite an abundance of robust evidence in favour of the Toxicofera clade, some still support old paradigms evidenced by morphological and behavioural evidence alone [10,11,12,13].

While helodermatid lizards have been considered venomous for over a century [14], applying the label ‘venomous’ to the oral secretions of other anguimorphs has faced resistance with the argument that toxic effects and their function in prey subjugation have been putatively assigned without evidence [13,14,15]. The Toxicofera controversy is further fuelled by arguments over the definition of ‘venom’ and ‘venom system’, more specifically questioning the activity of non-serpent venoms and the legitimacy of their venom system when compared to the highly derived venoms and venom systems of snakes [14,15]. In addition, many still endorse the ‘weaponised bacteria’ hypothesis for varanid lizards such as *V. komodoensis* and *V. giganteus* [16,17,18], despite a lack of evidence in favour of unique oral flora for predatory purposes [19].

The majority of lizard venom research has focused on members of the *Heloderma* genus due to severe envenomations with obvious ‘venom-like’ symptoms that have historically produced fatalities [14]. In addition to neurotoxins producing symptoms of immediate pain, convulsions and hemi-diaphragm paralysis, other toxins produce hypotension and promote bleeding through platelet blocking [14,20,21]. Previous studies have characterised the hypotensive activity of exendins [22,23,24], kallikrein enzymes [25,26,27,28], helokinestatins [1,22,29,30,31,32] derived from within the propeptide region of the hypotensive inducing B-type natriuretic peptides [1,22,29,31] found in the venoms of helodermatid lizards. Similar natriuretic peptides have also been found in other Anguimorpha that reduce arterial blood pressure via the relaxation of aortic smooth muscles [33]. Induced hypotension is an effective means of prey subjugation, with homologous natriuretic peptides found in the venoms of snakes such as *Oxyuranus microlepidotus* [34]. Similarly, many snake venoms promote bleeding by blocking platelet aggregation [35]. This mechanism of anticoagulation has been shown to be produced by GIII PLA_2_ enzymes from *H. horridum* venom and has been found to be present in other members of the genus [20,36].

The venom composition of helodermatid lizards has also been shown to be highly conserved despite speciation occurring up to 30 million years ago [36]. This is in contrast to many snakes that have developed different venom profiles even within the same species, likely due to a shift in primary prey either ontogenetically [37,38,39,40,41,42] or due to geographical location [43,44]. Therefore, similarities in the behaviour and prey of helodermatid lizards may contribute negative selection pressures resulting in their conserved venom profiles. Similarly, snakes such as *Notechis* that predominantly feed on the same prey taxa across their range have homologous venoms and functional activity despite occupying a broad geographical range [45].

In contrast to the conserved diet of helodermatid lizards, differences in diet across varanid species range substantially. While Australian dwarf monitors of the *Odatria* subgenus feeding mostly on invertebrates, frogs, smaller lizards and reptile eggs [46,47], larger species such as *V. giganteus* and *V. varius* consume anything they can overpower including mammals, birds, amphibians and smaller members of their own species [17,46]. The Komodo dragon, evolved to feed upon mammals in the 50 kg range [48], although it may on rare occasions successfully prey upon water buffalo which have been introduced into parts of this species’ range [49]. Other highly specialised species include *V. scalaris*, a small arboreal monitor that utilises tree hollows to ambush prey rather than the active foraging strategy that most *Varanus* species display [17]. Similarly, *V. prasinus* and relatives in the *Euprepriosaurus* subgenera are canopy specialists that seldom venture to the ground [46,50]. Whilst many of the smaller monitor species primarily feed on invertebrates, they are opportunistic predators that also feed on small vertebrates including mammals [50].

The activities of varanid lizard venoms have received little research attention, despite anecdotal evidence in the form of bite reports regularly describing profuse and persistent bleeding, clearly beyond the mechanical damage produced by the bite [1,51,52,53,54]. Indeed, many [13,18] are still sceptical of venom in varanids which may have deterred further investigation by other researchers. In contrast to the highly conserved venoms of helodermatid lizards, the venoms of varanid lizards have been shown to be extremely diverse and complex [54]. This is reflective of the range of ecological niches occupied by varanid lizards worldwide and suggests their venoms are under positive selection pressures facilitated by this diversification as the presence of coagulotoxic venom would be advantageous in the subjugation of warm-blooded vertebrate prey [35,41].

Previous studies have reported varanid lizard venom producing coagulotoxic activities comparable to that of the helodermatid lizards, including hypotension, promotion of bleeding through the blockage of platelets, and inhibition of blood coagulation [1,29,48]. Purified Group III PLA_2_ from the venom of *V. varius* was found to promote bleeding by inhibiting platelet aggregation via the same pathway as *Heloderma* venoms [1]. In addition, *V. komodoensis* and *V. varius* crude venoms were found to induce hypotension, with B-type natriuretic peptides found to be at least partly responsible for this activity [1,29,48]. However, varanids were shown to lack the gene for the helokinestatins, which are blood pressure acting toxins that evolved within the propeptide region of the hypotension inducing BNP peptides in the anguid/helodermatid last common ancestor after the split with the lanthanotid/varanid last common ancestor [29,33]. This is consistent with the proposed closer relationship of helodermatid lizards with the anguids and suggests active evolution amongst the Toxicofera clade.

Another possible pathway in which the anticoagulants could act is via the destructive cleavage of fibrinogen chains. The cleavage of fibrinogen by the enzyme thrombin is the last stage of the clotting cascade, producing a fibrin clot [55,56]. Each fibrinogen molecule is comprised of two symmetrical sets of three chains (Aα, Bβ, and γ) that are cleaved in a specific manner to expose polymerisation sites that allow for the crosslinking of fibrin [55,56]. Should an indiscriminate proteinase cleave fibrinogen in a non-specific way unlike thrombin, the ability of the fibrin chains to crosslink to producing a clot can be greatly reduced or prevented entirely. It has been shown in snake venoms that anticoagulation can be produced by either destructive, non-clotting cleavage of fibrinogen or a pseudo-procoagulant cleavage to produce a transient, weak clot that results in the consumption of fibrinogen [44,57,58]. Koludarov et al. [54] reported on the fibrinogenolytic activity of varanoid lizard venoms via gel electrophoresis. Whilst the helodermatid lizards displayed minimal activity despite the presence of proteolytic kallikreins [36], some varanid lizard venoms appeared to produce potent fibrinogenolysis [54]. However, though chains of fibrinogen were evidently cleaved, the functional ability of the venoms to reduce or prevent fibrin clots has not been ascertained and thus a knowledge gap exists.

Therefore, the aim of this study is to quantify the fibrinogenolytic activity of varanid lizard venoms via thromboelastography. By doing so, we will determine the functional effect of varanid venoms on the clotting ability of human fibrinogen in a more physiologically relevant system than has previously been used. Furthermore, we will investigate the role of co-factors in varanid lizard venom activity, which have been shown to be important catalysts in the activity of many coagulotoxic snake venoms [44,45,57,58,59].

## 2. Results

**Figure 1:**
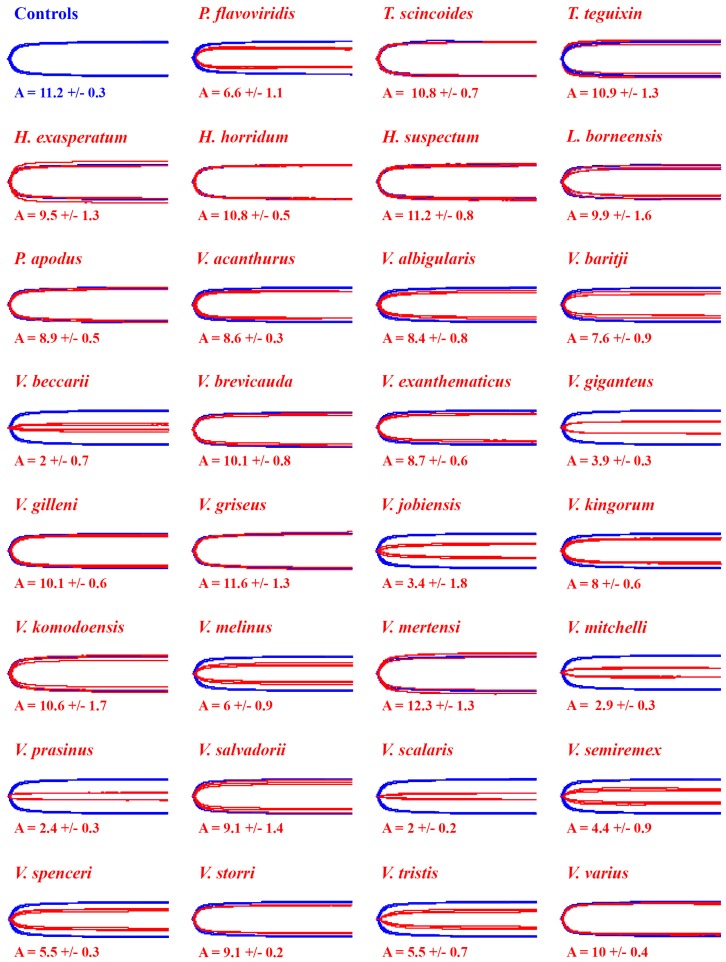
Overlaid thromboelastography traces showing effects of 1 μg/mL venoms (red traces) on ability to reduce fibrinogen clot formation relative to thrombin induced fibrin clot control (blue traces) with calcium/without phospholipid. A = amplitude of detectable clot strength (mm). Overlaid traces are *N* = 3 for each control or experimental condition. Values are means (*N* = 3) ± standard deviation.

**Figure 2:**
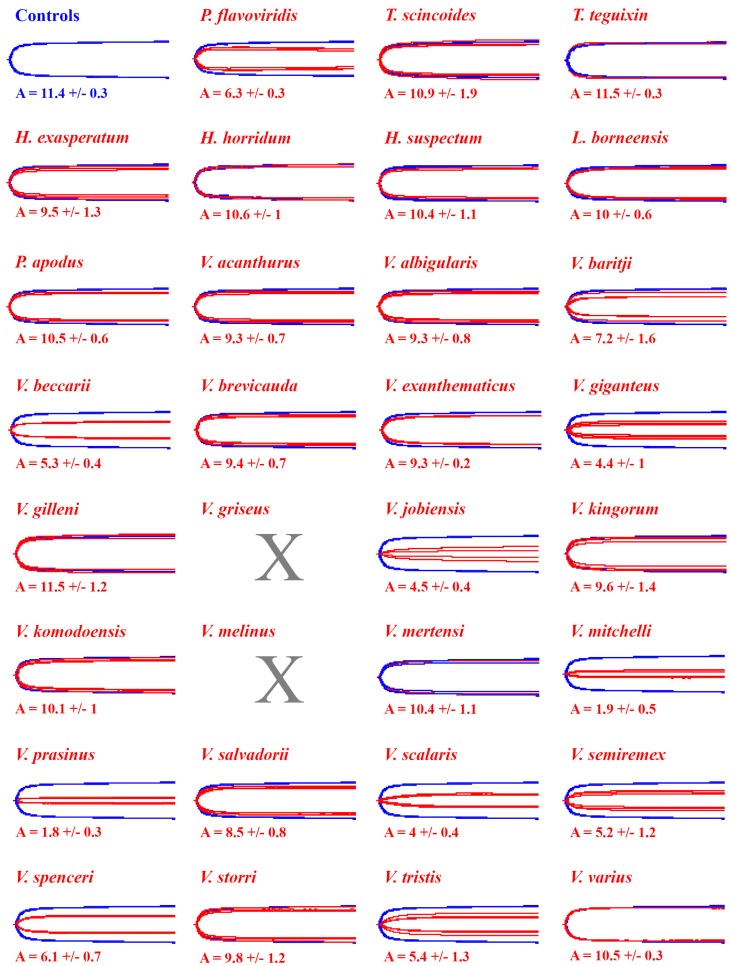
Overlaid thromboelastography traces showing effects of 1 μg/mL venoms (red traces) on ability to reduce fibrinogen clot formation relative to thrombin induced fibrin clot control (blue traces) with calcium/with phospholipid. A = amplitude of detectable clot strength (mm). Overlaid traces are *N* = 3 for each set of control or experimental conditions. *V. griseus* and *V. melinus* were excluded from the treatment as there were insufficient stocks. Values are means (*N* = 3) ± standard deviation. X indicates species which were not run due to lack of venom supply.

**Figure 3:**
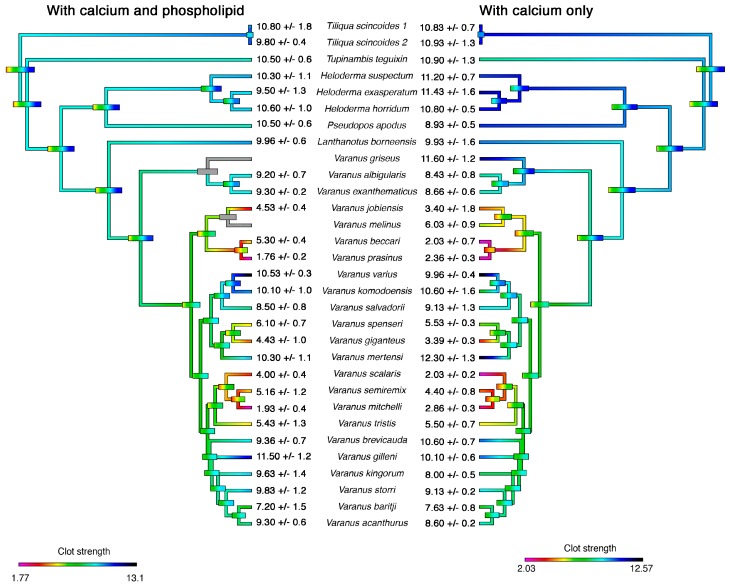
Ancestral state reconstructions of fibrinogenolytic activity from Figure 1 and Figure 2. Bars indicate 95% confidence intervals for the estimate at each node. Warmer colours indicate weaker clots (strong fibrinogenolytic activity) while cooler colours indicate stronger clots (weak fibrinogenolytic activity). Numbers at the tips are mean ‘A’ values ± standard deviation from *N* = 3 replicates. Phylogeny follows Ast (2001) [60] and Vidal et al. (2012) [61]. N.D. = not done due to lack of venom supply.

Testing revealed no difference in clot strengths when incubating the venoms in the presence or absence of calcium. However, relative presence or absence of the co-factor phospholipid was shown to exert an effect upon the relative toxicity of the venoms. The thrombin control for both treatments produced similarly strong clots with or without phospholipid (without PPL: 11.2 +/− 0.3; with PPL: 11.4 +/− 0.3) (Figure 1 and Figure 2). The three species of *Heloderma*, *L. borneensis* and *P. apodus* all produced clots within or close to control clot strengths and these venoms displayed no evidence of PPL dependence (Figure 1, Figure 2 and Figure 3). Similarly, saliva samples from the non-anguimorph, non-venomous lizard species (*T. scincoides* and *T. teguixin*) produced clots within control strengths as expected (Figure 1, Figure 2 and Figure 3).

In contrast to the undetectable activity of the *Heloderma* and *Lanthanotus* venoms at the concentrations tested in this assay, the fibrinogenolytic activities of varanid lizards were highly variable, including some demonstrating extremely potent activities with differential co-factor dependence (Figure 1 and Figure 2). Most species expressed minimal differences between co-factor dependence, with the exception of *V. beccarii* and *V. scalaris*, both of which were significantly more potent in the absence of PPL (*V. beccarii* P = 0.0017, *V. scalaris* P = 0.0014) (Figure 1 and Figure 2). The most potent species in the absence of PPL (*V. beccarii* (2 +/− 0.7 mm), *V. mitchelli* (2.9 +/− 0.3 mm), *V. prasinus* (2.4 +/− 0.3 mm) and *V. scalaris* (2 +/− 0.2 mm)) had stronger fibrinogenolytic activity than the positive control *P. flavoviridis* (6.6 +/− 1.1 mm), a pit viper from Okinawa, Japan with known fibrinogenolytic activity [57] (Figure 1). Other species within the activity range of *P. flavoviridis* or stronger include: *V. baritji* (7.6 +/− 0.9 mm), *V. giganteus* (3.9 +/− 0.3 mm), *V. jobiensis* (3.4 +/− 1.8 mm), *V. kingorum* (8 +/− 0.6 mm), *V. melinus* (6 +/− 0.9 mm), *V. semiremex* (4.4 +/− 0.9 mm), *V. spenceri* (5.5 +/− 0.3 mm) and *V. tristis* (5.5 +/− 0.7 mm) (Figure 1 and Figure 2). Clear phylogenetic patterns were evident, with destructive, non-clotting cleavage of fibrinogen amplified on at least three separate occasions: the *V. beccari*, *V. jobiensis*, *V. melinus*, and *V. prasinus* last common ancestor, with the lessened activity of *V. melinus* representing a secondary reduction; the *V. giganteus* and *V. spenseri* last common ancestor; and the *V. mitchelii*, *V. scalaris*, *V. semiremix* and *V. tristis* last common ancestor (Figure 3).

## 3. Discussion

We set out to quantify and determine the mechanism of Anguimorpha lizard venoms across 27 species. We found that fibrinogenolytic activity was amplified on at least three occasions within the varanid lizards (Figure 3) and that the multiple amplifications of this trait are strongly consistent with there being active evolution of these lizard venoms under positive selection pressure.

Our results are mostly consistent with the findings by Koludarov et al. 2017 [54], with a few exceptions. Interestingly it was the same multiple species from the 2017 study that cleaved the alpha and beta chains of fibrinogen relatively quickly that translated to reduced clotting ability in this study. This is evident in species such as *L. borneensis*, *V. acanthrus*, *V. baritji*, and *V. mertensi* that cleave the alpha chain quickly, but were slow to cleave the beta chain [54], translating to poor ability to reduce clot strengths in our study. An exception to this appears to be *V. giganteus* that, despite slow onset beta chain cleavage, still significantly reduced the clotting ability of human fibrinogen in this study (Figure 1, Figure 2 and Figure 3). Therefore, our study demonstrates the importance of using more physiologically relevant functional testing such as thromboelastography over techniques such as fibrinogen gels that only imply activity [54]. Future studies could investigate degradation products further by looking into the cleavage sites and degradation products of fibrinogen by the different venoms. The variability in target cleavage sites could explain why *V. giganteus* still maintains fibrinogenolytic activity while other alpha chain cleavers do not.

Co-factor testing revealed that phospholipid has an important role in the activity of some lizard venoms, such as reducing action in *V. beccarii* and *V. scalaris* by half when present (Figure 1 and Figure 2). Interestingly, both the sister species of *V. beccarii* (*V. prasinus*) and *V. scalaris* (*V. mitchelli*) did not express reductions in activity, suggesting the trait may have arisen independently in the two clades. Future work should investigate the structural variations in the venom enzymes and how this affects the relative co-factor dependence and utilisation.

The phylogenetic pattern of fibrinogenolytic activity revealed by our results suggests the trait is actively selected for under positive selection pressures (Figure 3). This toxic effect, while non-lethal, coupled with other previously described activity in conjunction with mechanical damage from the teeth, could aid in the subjugation of the lizard’s prey. The ability to weaken prey items enough to consume it would be equally selected and as effective a method for gaining a meal as killing the prey, as the same ultimate goal is achieved [62]. Thus, fibrinogenolytic activity could have a predatory role in the venoms of varanids by facilitating blood loss initiated by the mechanical damage inflicted by their large teeth and violent head movements, and therefore be a part of the combined predatory arsenal.

The potent activity seen in *V. beccarii*, *V. prasinus*, and *V. scalaris* may be particularly strongly selected due to their arboreal nature. Should prey escape from these monitors by falling to the ground or flying away, it is unlikely to be consumed, as these arboreal species are reluctant to venture to the ground. Therefore, arboreal monitors are under strong selection pressure to subjugate prey quickly or to substantially weaken escaped prey in order to be recaptured swiftly. Potent toxic activity has been observed in other arboreal reptiles under similar selection pressures, such as the elapid snake *Dendroaspis angusticeps* [63] and colubrid snakes such as *Dispholidus typus* and *Thelatornis mossambicanus* [64]. Similarly, cone snails (*Conus*) that hunt fish have developed rapid acting venoms to subdue their highly mobile fish prey [65]. Thus, escape potential appears to play a major role in venom evolution across venomous taxa.

Desert and plain dwelling monitors such as *V. giganteus* and *V. spenceri* may be under similar selection pressures for amplified activity, as they occupy arid landscapes with prey densities that fluctuate in a “boom-bust” state. In these environments lost prey can lead to death, especially in drier seasons when prey is less abundant and often one of few sources of moisture [66,67]. In this scenario, fibrinogenolytic activity post bite, coupled with other toxic effects and mechanical damage, could kill or weaken any escaped prey, allowing the monitor to follow the scent to an easy meal. Monitor lizards have acute chemosensory abilities and are capable of tracking the scent of prey from considerable distances [17,68]. Therefore, despite the majority of predation observations ending quickly and violently [13], should the prey escape, toxic activities that weaken the quarry for recapture would be advantageous and therefore positively selected.

Other possible functions of fibrinogenolytic activity in varanids is in digestion or defence [13,47]. This venom activity could be the result of indiscriminate proteolytic enzymes that may aid in digestion. However, the phylogenetic pattern seen in our results does not reflect arbitrary activity with a digestive role (Figure 3). Stimulating pain is an effective predator deterrent adopted by many animal venoms [35]. However, fibrinogenolytic activity is not painful. Thus, the biochemical modes of action suggest a predatory role for the venom enzymes that destructively cleave fibrinogen.

The present study is the first to demonstrate the functional ability of lizard venoms to reduce clot formation and the first to investigate the role co-factors play in the activity of lizard venoms. Our results are consistent with anticoagulant symptoms of bite reports from varanids and identifies at least one potent anticoagulant pathway. The variability of fibrinogenolytic activity within the varanids suggests the trait is under active selection pressure and supports a functional role of anguimorph lizard venom in prey subjugation. This represents adaptive molecular evolution of an enzyme (salivary kallikrein) repurposed for a new function (destructive cleavage of fibrinogen). This is consistent with the modes of evolution proposed for reptile venoms in which endogenous proteins are co-opted for selective use in the venom [69]. These activities are distinct from those recently described for *Heloderma* venoms which had a procoagulant effect [70].

Kallikrein enzymes in snakes, similar to the toxins likely responsible for the fibrinogenolytic activity in lizards [54], have therapeutic applications in stroke and other coagulation related disorders [71]. Furthermore, a toxin from *H. suspectum* has already been modelled for a wide therapeutic used to treat people suffering from type 2 diabetes [72]. Therefore, as a neglected lineage of venomous organism, lizard venoms could provide a new avenue for biodiscovery. In addition, future work should investigate other anticoagulant mechanisms of lizard venoms such as inhibition of clotting enzymes.

## 4. Materials and Methods

### 4.1. Venom Samples

The majority of Australian lizard species samples were obtained during a transcriptome study [29] under the University of Melbourne (2005) approval UM0706247. Venoms were collected by encouraging the specimens to chew on soft rubber tubing, with the mandibular secretions collected with pipettes, centrifuged at 14,000 RCF (4 °C) to remove insoluble material, filtered with 40 micron syringe filters to remove mucous, flash frozen in liquid nitrogen, and then freeze dried. These samples include: *Varanus acanthurus* (Newman, WA, Australia), *V. baritji* (Adelaide River, NT, Australia), *V. giganteus* (Sandstone, WA, Australia), *V. mitchelli* (Kununurra, WA, Australia), *V. scalaris* (Kununurra, WA, Australia), *V. komodoensis* (Singapore Zoo, captive specimen, unknown founding locality) and *V. varius* (Mallacoota, VIC, Australia). The remaining Australian samples were obtained through private and commercial collections under the University of Queensland (2016-2019) ethics approval SBS/403/16. These samples include: *Tiliqua scincoides* (captive specimen, unknown founding locality), *V. brevicauda* (captive specimen, unknown founding locality), *V. gilleni* (captive specimen, Alice Springs, NT, Australia founding locality), *V. kingorum* (captive specimen, Turkey Creek, WA, Australia founding locality), *V. mertensi* (captive specimen, Kununurra, WA, Australia founding locality), *V. prasinus* (captive specimen, unknown founding locality), *V. semiremex* (captive specimen, Cairns area, QLD, Australia founding locality), *V. spenceri* (captive specimen, Barkley Tableland, NT, Australia founding locality), *V. storri* (captive specimen, unknown founding locality) and *V. trisitis* (captive specimen, Western QLD, Australia founding locality). Non-Australian lizard samples were obtained from captive specimens from unknown founding localities by Alphabiotoxine Laboratory, Montroel-au-bois, Belgium. These samples include: *Tupinambis teguixin*, *Heloderma exasperatum*, *H. horridum*, *H. suspectum*, *Lanthanotus borneensis*, *Pseudopus apodus*, *V. albigularis*, *V. beccari*, *V. exanthematicus*, *V. griseus*, *V. jobiensis*, *V. melinus* and *V. salvadorii.* Okinawa Habu Pit-Viper *Protobothrops flavoviridis* was used as a positive control (captive specimen, Okinawa, Japan founding locality), as it is known to have fibrinogenolytic activity [57].

### 4.2. Thromboelastography

The ability of Anguimorpha lizard venoms to reduce clot strength of human fibrinogen was measured using a Thromboelastograph^®^ 5000 Haemostasis analyser (Haemonetics^®^, Haemonetics Australia Pty Ltd., North Rdye, Sydney, Australia) as previously described [44,57,58,73]. Human fibrinogen (Lot#F3879, Sigma Aldrich, St. Louis, MO, USA) was reconstituted in enzyme running buffer (150mM NaCl, 50mM Tri-HCl (pH 7.3)) to a concentration of 4 mg/mL. Natural pins and cups (Lot# HMO3163, Haemonetics Australia Pty Ltd., North Rdye, Sydney, Australia) were used maintaining the same stoichiometry as for the clotting time tests (see Section 4.1). Volumes were proportionally changed to accommodate the larger reaction volume: 7 μL of the 1 mg/mL venom working stock (lyophilised venom in 50% glycerol/50% deionised water) or 7 μL 50% glycerol/50% deionised water for negative control, 72 μL CaCl_2_ (25mM stock solution Stago Cat# 00367 STA), 72 μL phospholipid (solubilized in Owren Koller Buffer adapted from STA C·K Prest standard kit, Stago Cat# 00597) when conducting PPL co-factor dependence assays (volume replaced with Owren Koller Buffer for assays without PPL), and 20 μL Owren Koller Buffer (Stago Cat# 00360) (92 μL without PPL) was combined with 189 μL human fibrinogen, pipette mixed and incubated for 30 min at 37 °C. Fibrin clots were then induced by adding 7 μL of thrombin (stable thrombin from Stago Liquid Fib kit, unknown concentration from supplier (Stago Cat#00673 Liquid Fib)) and run for a further 30 min to determine the extent of the fibrinogenolysis occurring. Clot strength was determined by the output parameter ‘A’ (Amplitude) defined as the width of the trace at the last time point and is measured in mm. All experiments were run in triplicate unless venom stocks were depleted. Other output measurements (e.g., split point; angle) were not analysed because were uninformative for these venoms.

### 4.3. Figures and Analysis

The phylogenetic tree (adapted from [60,61]) was produced using Mesquite software (version 3.2) [74] and then imported to Rstudio using the APE package [75]. Ancestral states were estimated for all traits using maximum likelihood as implemented in the contMap function of the R package phytools [76]. Statistical significance between treatment types was determined by Student’s *t*-test.
